# Topical application of FTY720 and cyclosporin A prolong corneal graft survival in mice

**Published:** 2012-03-09

**Authors:** Yong Liu, Jingjing Jiang, He Xiao, Xiaokui Wang, Yan Li, Yubo Gong, Dajiang Wang, Yifei Huang

**Affiliations:** 1 Departments of Ophthalmology, Chinese PLA General Hospital, Beijing, China; 2 Department of Ophthalmology, Chinese PLA Air force General Hospital, Beijing, China; 3 Department of Molecular Immunology, Institute of Basic Medical Sciences, Beijing, China; 4 Department of Molecular Drug Design, Institute of Pharmacology and Toxicology Sciences, Beijing, China

## Abstract

**Purpose:**

To investigate the effects of topical FTY720 and cyclosporin A (CsA) on allogeneic corneal transplantation in mice.

**Methods:**

A total of 75 BALB/c mice received corneal grafts from C57BL/6 donors. Recipients were treated with 0.1%, 0.3%, or 0.5% FTY720 ophthalmic gel or 1% CsA eye-drops after the graft (controls received no treatment). The number of cluster of differentiation (CD)4+ T cells and CD4+CD25+forkhead box P3 (Foxp3)+ regulatory (Treg) cell phenotypes were measured by flow cytometry. Cytokine mRNA expression in corneal grafts was analyzed by real-time quantitative PCR. CD4 + T cells and cytokines in corneal samples were identified by immunohistochemical staining.

**Results:**

Corneal graft survival was prolonged by treatment with topical 0.5% FTY720 (mean survival time [MST], 24.1±1.6 days) or 1% CsA eye-drops (MST 25.0±1.9 days) compared with controls (MST, 13.4±0.5 days; n=9, both p<0.01). Topical 0.5% FTY720 treatment significantly increased the percentages of CD4 + T (p<0.05) and Treg cells (p<0.01; n=5) in the cervical lymph nodes compared with controls. Transforming growth factor-β1 (*TGF-β1*) mRNA transcription in corneal grafts after topical 0.5% FTY720 increased (p<0.05, n=3), while interleukin-2 (*IL-2*) and interferon-γ (*IFN-γ*) mRNA expression in corneal grafts treated with 1% CsA decreased (p<0.01, p<0.05, respectively). These cytokine results were paralleled by similar immunohistochemical staining. Topical 0.5% FTY720 and 1% CsA treatment reduced the infiltration of CD4+ Tcells in the grafts.

**Conclusions:**

Topical 0.5% FTY720 and 1% CsA can effectively prolong allogeneic corneal graft survival in mice. Treatment with topical 0.5% FTY720 increases the percentage of CD4+ T cells and the percentage of Treg cells in cervical lymph nodes. The 0.5% FTY720 increased *TGF-β1* mRNA expression and decreases infiltration of CD4+ T cells in corneal grafts, while topical 1% CsA down-regulated the expression of *IL-2* and *IFN-γ*.

## Introduction

Corneal diseases are the second most important cause of blindness [1]. Some of these cornea conditions, such as inflammation or infection, can be treated with medication. Severe cases that are not treatable with medication, or in cases where there is scarring or cloudiness of the cornea that results in visual loss, may require corneal transplantation to improve vision. Corneal transplantation, known as penetrating keratoplasty, is one of the most common and successful forms of solid organ transplantation in humans [2]. Williams et al. reported that the probability of penetrating corneal graft survival in the whole cohort was 87%, 73%, 60%, and 46% at 1, 5, 10, and 15 years respectively [3]. In addition, in high-risk grafts that have either received a previous corneal transplant or prevascularized graft beds, the success rate fell dramatically to be as low as 20% to 40% [4-6]. Irreversible immune rejection of the transplanted cornea is the major cause of human allograft failure in the intermediate and late postoperative period [7]. Corneal graft-rejection is a complex immune process consisting of a sequence of events. Corneal allograft rejection requires the presence of T lymphocytes (likewise called T cells), and cluster of differentiation (CD)4+T cells are the most important T cell population. Additionally, two ocular antigen presenting cell populations — corneal Langerhans cells and conjunctival macrophages — are also required [8]. In recent years, some studies reported that corneal allograft survival was associated with CD4+CD25+forkhead box P3 (Foxp3)+ T regulatory (Treg) cells [9,10].

Currently available immunosuppressive drugs, such as corticosteroids and cyclosporin A (CsA), are used to prevent or treat corneal graft rejection in humans, but long-term survival of corneal grafts, especially in high-risk recipients, have not been entirely efficacious [11,12]. As a synthetic structural analog of myriocin, FTY720 is a potent immunosuppressant that can prolong allograft survival [13]. Once it is phosphorylated in vivo by sphingosine kinase 2 (SphK2), FTY720-P acts as an agonist on four of the five known sphingosine-1-phosphate (S1P) receptors (S1P1, S1P3, S1P4, and S1P5) [14]. In contrast to classical immunosuppressants, it has been shown that FTY720 does not interfere with T-cell proliferation, but induces a severe deprivation of lymphocytes in the blood due to modification of S1P signaling [15]. A multicenter, dose-finding study compared the effectiveness of FTY720 plus full-dose CsA (FDC), FTY720 plus reduced-dose CsA (RDC), and mycophenolate mofetil (MMF) plus FDC in de novo renal transplant patients. The study found that 5 mg FTY720 in combination with RDC or 2.5 mg FTY720 combined with FDC showed comparable rejection prophylaxis and acceptable tolerability at 12 months compared with MMF plus FDC [16]. Recently, Li et al. [17] found that the single use of FTY720 can be effective to prolong the graft in rat cardiac transplantation model. Sedláková et al. [18] found that intraperitoneal injections of FTY720 could prolong graft survival in rat-to-mouse corneal xenografts. Other two studies also found that oral immunosuppression with FTY720 significantly prolonged corneal allografts [19,20]. However, systemic FTY720 treatment was reported to cause nonfatal herpesvirus infections, bradycardia and atrioventricular block, hypertension, macular edema, skin cancer, and elevated liver-enzyme levels [21]. To avoid the side-effects of systemically administered immunosuppressant, we tested the topical use of FTY720 and CsA in a mouse allogeneic corneal transplantation model over a one- month period.

## Methods

### Animals

Orthotopic corneal transplantation was performed with inbred BALB/c and C57BL/6 male mice. Mice used in grafting experiments weighed 18–22 g. C57BL/6 mice served as donors and BALB/c mice were recipients of the corneal allograft. These are fully mismatched for major histocompatibility complex and multiple minor histocompatibility antigens between the two inbred mouse strains. Mice were obtained from the Beijing HFK Bio-Technology. Co., Ltd. Beijing, China. All animals were treated in accordance with the Association for Research in Visio and Ophthalmology Statement on the Use of Animals in Ophthalmic and Vision Research.

### Orthotopic allogeneic corneal transplantation

A total of 75 BALB/c mice received corneal grafts from C57BL/6 donors were randomized into a control group and four experimental groups. Before all surgical procedures, mice were deeply anesthetized by an intraperitoneal injection of 3% pentobarbital sodium (80 mg/kg; Nembutal; Beijing Chemical Co., Beijing, China). A tropicamide-phenylephrine ophthalmic solution (Santen Pharmaceutical Co., Ltd, Japan) was topically applied to dilate the pupil of both the donors and recipients. The donor cornea was prepared according to the “underwater technique” originally described by Zhang et al. [22]. The central 2.0 mm of a C57BL/6 cornea was marked with a 2.0 mm trephine, excised with Vannas scissors and placed into a balanced salt solution (BSS™; Alcon, Fort Worth, TX) before grafting. The donor corneal graft was sutured into a 1.5 mm BALB/c recipient corneal bed with 8–10 interrupted 11–0 nylon sutures (Sharpoint, Reading, PA). The anterior eye chamber was restored at the end of surgery by injecting air. Corneal sutures were removed on day 10 after transplantation. Mice that received in situ ophthalmic gel without any drugs (i.e., only a gel base) served as controls. One experimental group of mice received CsA eye-drops (10 mg/ml; 1%; North China Pharmaceutical Group Co., Ltd. Hebei, China) after transplantation. Other three experimental groups of mice were treated in situ with an ophthalmic gel of FTY720 (provided by the Department of Molecular Drug Design, Institute of Pharmacology and Toxicology Sciences, Beijing, China.) at doses of 1 mg/ml (0.1%), 3 mg/ml (0.3%), and 5 mg/ml (0.5%), respectively, after transplantation. Ophthalmic gel was applied twice a day and eye-drops administered four times a day from day 0 (post-operation) to day 30 (the end of study).

### Clinical evaluation of grafted corneas

The degree of opacity as well as the degree of neovascularization was evaluated daily until day 14 and then three times a week for the remaining two weeks. Briefly, donor corneal opacity score (0–4), edema score (0–2), and neovascularization score (0–4) were graded according to criteria previously described [23]. Rejection was defined as the day on which indices of opacity, edema, and neovascularization reached moderate or severe levels, with an opacity score ≥3 and a total ≥5, in grafts that were initially transparent [23]. Grafts with technical difficulties such as intraocular hemorrhage or infection were excluded. Six mice in each group were sacrificed for laboratory examination on day 14 after transplantation. An additional nine mice in each group were observed for survival time. At the time of rejection or the end of the study, mice were sacrificed.

### Flow cytometric analysis

The T cell phenotype in the right cervical lymph node, peripheral blood, and the spleen of five mice in each group were analyzed by flow cytometry on day 14 after transplantation. Treg cells were detected by a Mouse Regulatory T cell Staining Kit (PE Foxp3 FJK-16s, FITC CD4, APC CD25) from eBioscience (San Diego, CA), according to the manufacturer's instructions. Data was acquired using a FACS Calibur flow cytometer (BD Biosciences, San Jose, CA), and analyzed with Winmid 2.9 software (Scripps Institute, La Jolla, CA). The cells were gated on lymphocytes, further gated for CD4+, and are shown as the percentage of CD4+ T cells and CD4 + CD25 + FoxP3 +T cells.

### Real time quantitative PCR

Three corneas in each group were excised (2.5 mm in diameter), frozen in liquid nitrogen, and stored at −80 °C on day 14 after transplantation. For intragraft gene expression analysis, total cellular RNA was isolated using the TRIzol® reagent (Invitrogen, Carlsbad, CA) and liquid nitrogen. Reverse transcription of mRNA to cDNA was performed in 20 μl reaction volumes with random priming and EasyScript RT using Easy RT–PCR Kit (Beijing TransGen Biotech Co. Ltd, Beijing, China). Gene expression was examined in an iCycler IQ Real-time PCR Detection System (Bio-Rad, Hercules, CA) using the SYBR Green Realtime PCR Master Mix (TOYOBO, Osaka, Japan) with respective real-time quantitative PCR (qPCR) primers for interleukin-2 (*IL-2*), *IL-10*, transforming growth factor-β1 (*TGF-β1*), *Foxp3*, and glyceraldehyde-3-phosphate-dehydrogenase (*GAPDH*) [24,25]. The primers used are listed in Table 1. The cycle number at which the reporter fluorescence reached a threshold (CT value) was used for quantitative measurement. The relative expression data was determined by normalizing to *GAPDH* expression measured contemporaneously from the same sample to calculate a fold-change in value using the 2^−ΔΔCT^ method.

**Table 1:** Primer sequences used for qPCR.

**Gene**	**Primer 1 (Forward, 5′→3′)**	**Primer 2 (Reverse, 5′→3′)**
*GAPDH*	TGAAGGTCGGTGTGAACGGATTTG	GTTGAATTTGCCGTGAGTGGAGTC
*IL-2*	GCACCCACTTCAAGCTCCA	AAATTTGAAGGTGAGCATCCTG
*IL-10*	TGCCTTCAGCCAGGTGAAGACTTTC	CTTGATTTCTGGGCCATGCTTCTCTG
*TGF-β1*	ATACCAACTATTGCTTCAGCTCCACAG	GTACTGTGTGTCCAGGCTCCAAATAT
*IFN-γ*	GCACAGTCATTGAAAGCCTAGAAAGTC	GGTAGAAAGAGATAATCTGGCTCTG
*Foxp3*	ATGCCCAACCCTAGGCCAGCCAAG	TGGGCCCCACTTCGCAGGTCCCGAC

### Histopathological examination

Fourteen days after transplantation, the other three corneas in each group were fixed in 10% formaldehyde solution, paraffin-imbedded, and sectioned at 3 µm. Slides were deparaffinized and stained with hematoxylin and eosin for histological examination of the pathology, or processed for immunohistochemical analysis. After deparaffinization, the slides were rehydrated, and heat-induced antigen retrieval was performed. Then immunostaining was performed using the following primary antibodies, Anti-CD4 Mouse monoclonal (mAb51312; Abcam, Cambridge, UK), anti-IFN Gamma (Biorbyt, Carrickfergus, UK), IL-2, IL-10, and TGF-β1 (Santa Cruz Technologies, Santa Cruz, CA); a horseradish peroxidase -conjugated goat anti-mouse immunoglobulin G (H^+^L) (Zymed Laboratories, San Francisco, CA) was used as the secondary antibody. Incubation with the diaminobenzidine (DAB) chromogen for 5 min was used to visualize positive staining (brown staining) followed by counterstaining with Mayer’s hematoxylin for 1 min. PBS replaced the primary antibody as a negative control. Photographs of the same anatomic area- the central zone of each graft of each slide were used for comparison. The positive stained cells were counted on these the photographs.

### Statistical analysis

Actuarial graft survival was analyzed with the Kaplan–Meier survival method, and the log-rank test was used to examine statistical differences among the groups. A One-way ANOVA followed by multiple comparisons with least significant difference (LSD) test was used in all other cases. A p value <0.05 was considered significant.

## Results

### Orthotopic allogeneic corneal transplantation

**Figure 1:**
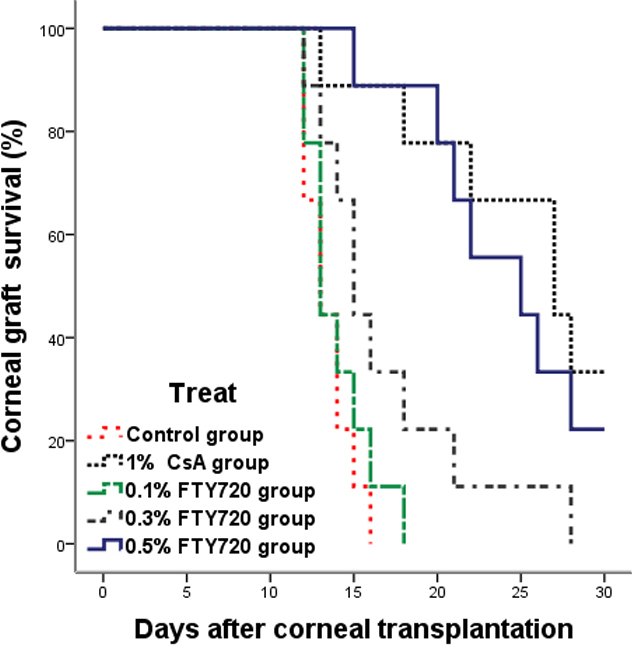
Prolongation of corneal allograft survival by topical administration. Allograft survival of the control versus topical 0.1% FTY720 were not significantly different (p=0.47; n=9) but was compared with the topical 0.3% FTY720 treatment group (p=0.03; n=9). Treatment with 0.5% FTY720 or 1% CsA also resulted in significant graft survival compared to controls (both p<0.01; n=9) or 0.3% FTY720 treatment (p=0.02 and p=0.01, respectively). There was no significant difference between 0.5% FTY720 and 1% CsA (p=0.51).

**Figure 2:**
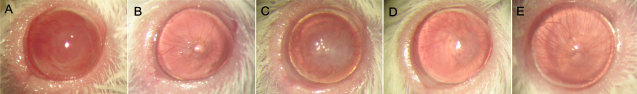
Opacity, edema and neovascularization of corneal grafts were observed. **A**: Ccontrol group on postoperative day 14. B: Topical 1% CsA group on postoperative day 30. **C**: Topical 0.1% FTY720 group on postoperative day 14. **D**: Topical 0.3% FTY720 group on postoperative day 14. **E**: Topical 0.5% FTY720 group on postoperative day 30.

Transplantation of C57BL/6 corneal grafts to BALB/c recipients resulted in a rejection rate of 100% within a mean survival time (MST) of 13.4±0.5 days (n=9) in the control group, which was not statistically significant different from the 0.1% FTY720 ophthalmic gel group (MST 14.0±0.7 days; p=0.47; Figure 1). Although the rejection rate was also 100% with 0.3% FTY720 treatment (MST 16.9±1.7 days), graft survival was prolonged when compared with the control group (p=0.03). Treatment with either 0.5% FTY720 (MST 24.1±1.6 days) or 1% CsA (MST 25.00±1.91 days) significantly prolonged the period before rejection compared to 0.3% FTY720 gel treatment (p=0.02, 0.01, respectively, versus control p<0.01) and did not reach 100% rejection within the 30 days. The corneal allografts being rejected exhibited pronounced opacity, edema and neovascularization on postoperative day 14 (Figure 2A,C,D). As shown in Figure 2B,E, the corneal allografts seen after treatment with 1% CsA or 0.5% FTY720 exhibited a clear stroma at the end of study.

### Flow cytometric analysis

**Figure 3:**
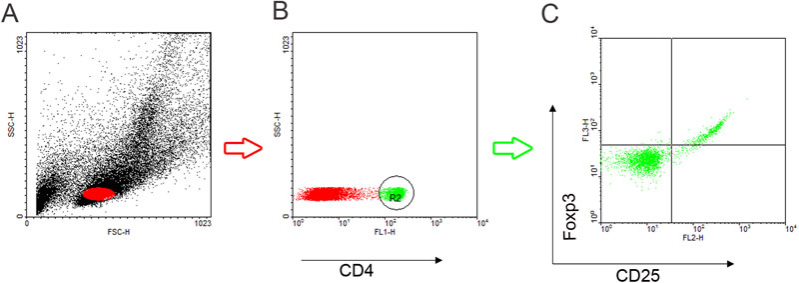
Flow cytometric analysis of the T cell phenotype in the right cervical lymph node, peripheral blood, and spleen. **A**: Flow cytometry of the spleen of control mouse 2 on postoperative day 14. Gate 1: lymphocytes (R1, indicated in red). **B**: CD4+T cells (R2. Indicated in green) in lymphocytes. **C**: Double labeling CD25+/Foxp3+ lymphocytes in CD4+ T population. Gate 2: R1 and R2.

**Figure 4:**
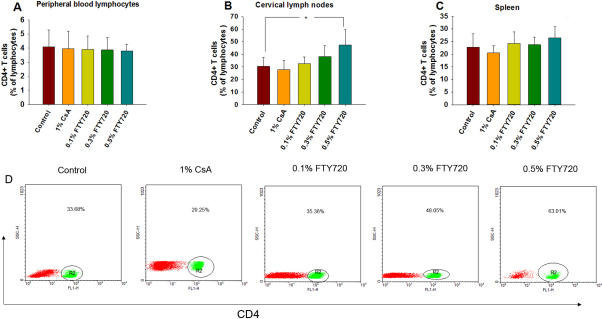
The percentage of CD4+ T cells in the lymphocyte population in each group (%,) on postoperative day 14. The percentage of CD4+ T cells in the peripheral blood lymphocyte population (**A**), cervical lymph nodes (**B**), and spleen (**C**). Values represent mean±SD, n=5 mice/group, *p<0.05 versus the control group. D: Flow cytometry showing the distribution of CD4+ T cells in lymph nodes.

**Figure 5:**
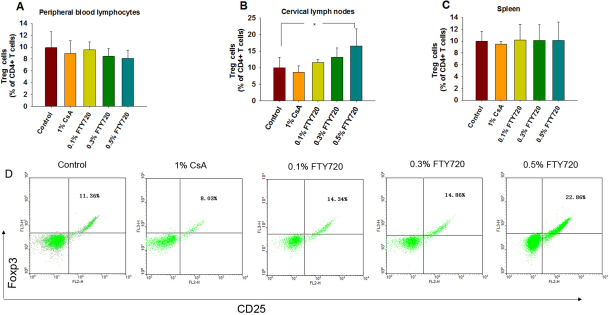
The percentage of CD4+CD25+Foxp3+ T (Treg) cells in the CD4+ T cell population in each group (%,) on postoperative day 14. The percentage of Treg cells in the peripheral blood lymphocyte population (**A**), cervical lymph nodes (**B**), and spleen (**C**). Values represent mean±SD, n=5 mice/group, *p<0.05 versus the control group. **D**: Flow cytometry showing the distribution of double CD25+Foxp3+ T cells in the CD4+ T cell population in the lymph nodes.

Figure 3 shows an illustrative example of how the determination was performed by flow cytometry data. There were no significant differences in the mean percentage of CD4 + T cells in the peripheral blood samples (peripheral blood lymphocytes, PBLs) or the spleens of mice within the five groups (p>0.05, n=5; Figure 4A,C). Similarly, there were no statistical differences among the five groups (p>0.05, Figure 5A,C) in the mean percentage of CD4+CD25+Foxp3+ T cells in the CD4 + T population of the PBLs or spleen. The mean percentage of CD4 + T cells in cervical lymph nodes of the 0.5% FTY720 ophthalmic gel group was higher than the control group and 1% CsA group, respectively (p=0.04 and p=0.02, respectively, n=5; Figure 4B,D). There were also significantly higher percentages of CD4+CD25+ Foxp3+ T cells in CD4+ T population in cervical lymph nodes after 0.5% FTY720 treatment versus the control group or 1% CsA group respectively (both p<0.01, Figure 5B,D).

### Real-time quantitative PCR

**Figure 6:**
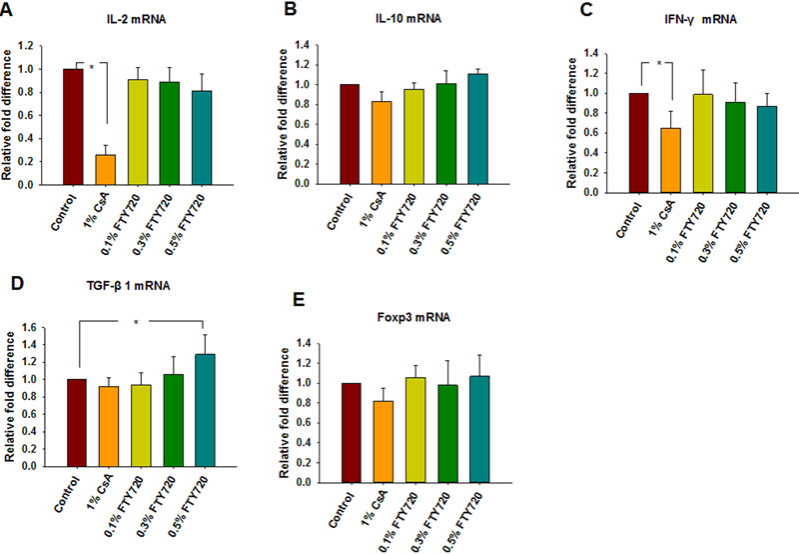
The relative changes in cytokine mRNA expression in the corneal grafts. Values are given relative to an increase or decrease from the control value in each group (mean±SD, n=3 mice/group) at postoperative day 14. *p<0.05 versus the control group. (**A**) *IL-2*, (**B**) *IL-10*, (**C**) *INF-γ*, (**D**) *TGF-β1*, (**E**) *Foxp3*.

The results of mRNA expression in corneal grafts are shown in Figure 6. When compared with the control group, real-time quantitative PCR analysis demonstrated a significant reduction of *IL-2* and *IFN-γ* mRNA expression following topical application 1% CsA (p<0.01 and p=0.04, respectively, n=3; Figure 6A,C). Although the levels of *IL-10* mRNA expression in the corneal graft after topical 0.3% and 0.5% FTY720 treatment were slightly higher than those seen in the control group, there were no statistical differences among the five groups (p>0.05, n=3; Figure 6B). Compared with the control group, *TGF-β1* mRNA transcription in corneal grafts with topical 0.5% FTY720 increased (p=0.04, Figure 6D). An increase in *Foxp3* mRNA expression in the corneal grafts of the 0.5% FTY720 treatment group was observed, but differences among the five groups were not statistically significant different (p>0.05, Figure 6E).

### Histopathological examination

**Figure 7:**
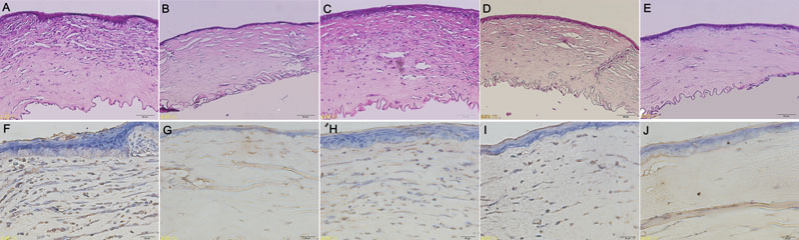
Histopathology of corneal grafts. Examples of grafts stained with hematoxylin-eosin on postoperative day 14 (**A-E**, Magnification, 20×). **A**: Untreated corneal graft (control). **B**: Topical application of 1% CsA. **C**-**E**: Topical application of 0.1% 0.3% or 0.5% FTY720, respectively. Examples of grafts showing CD4 positive staining (brown) on postoperative day 14 (**F**-**J**, Magnification, 40×). **F**: Untreated corneal graft (control). **G**: Topical application 1% CsA. **H**-**J**: Topical application of 0.1% 0.3% or 0.5% FTY720, respectively.

**Figure 8:**
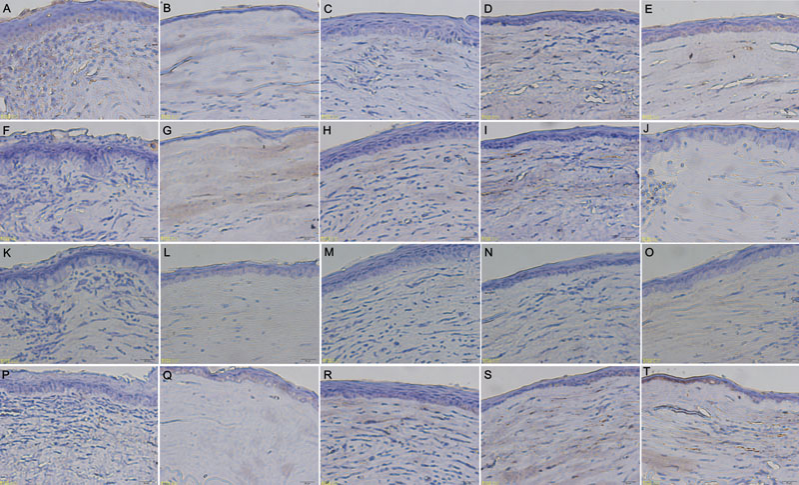
Cytokine Immunohistochemical staining in corneal grafts on postoperative day 14. **A**, **F**, **K**, and **P**: Untreated corneal graft (control). **B**, **G**, **L**, and **Q**: Topical 1% CsA group. **C**, **H**, **M**, and **R**: Topical 0.1% FTY720 group. **D**, **I**, **N**, and **S**: Topical 0.3% FTY720 group. **E**, **J**, **O**, and **T**: Topical 0.5% FTY720 group. The brown staining represents positive IL-2 protein (**A**-**E**), positive IL-10 protein (**F**-**J**), positive IFN-γ protein (**K**-**O**), and positive TGF-β1 protein (**P**-**T**).

Histopathological analysis of the enucleated formalin fixed corneal grafts of the BALB/c mice showed signs of graft rejection in the five groups 14 days after corneal transplantation (Figure 7). The allografts in the control and topical 0.1% FTY720 groups (Figure 7A,C, respectively) revealed a heavy infiltration of inflammatory cells, edema, and disruption of the stromal architecture. There was less infiltration of inflammatory cells, and edema following the topical application of 1%CsA or 0.5% FTY720 (Figure 7B,E, respectively) compared with the control group. Immunohistochemical staining showed that a large number of CD4+ T cells (brown) had infiltrated the allograft in control and topical 0.1% FTY720 groups (Figure 7F,H, respectively). In contrast, no significant infiltration of CD4+ cells was observed in allografts after topical application of 1% CsA or 0.5% FTY720 (Figure 7G,J, respectively). In agreement with the results of mRNA expression, the levels of IL-2 and IFN-γ protein immunohistochemical staining in corneal grafts were reduced after topical application of 1% CsA (Figure 8B-L). The immunohistochemical staining results indicated that TGF-β1 protein in the allografts after topical 0.5% FTY720 was higher than that seen in control group (Figure 8P,T). There were no apparent differences in the staining levels of IL-10 protein in the allografts among the five groups (Figure 8F-J).

## Discussion

Corneal allotransplantation is the most common form of solid tissue transplantation in humans and is characterized by a high success rate of graft survival, but immunological rejection remains a serious risk factor for corneal graft failure [4-8,26]. Immunosuppressants, such as CsA and FK506, are now used for prevention of allograft rejection in clinical corneal transplantation. Although systemic CsA has clear therapeutic efficacy, it is limited by the potential systemic side-effects [27]. Topical CsA has been used and studied extensively with regard to the management of corneal graft rejection in recent years, but there were still some contradictory results [28-32]. As a new immunosuppressant, systemic treatment with FTY720 can prolong corneal graft survival, but there are some side-effects [16-18,21]. Nevertheless, FTY720 can be dissolved in DMSO and water [33], and can also be used for topical application.

We found that 0.5% FTY720 ophthalmic gel can effectively prolong the survival of mouse corneal allografts. Although Unal et al. [32] and Poon et al. [30] reported that topical 0.05% CsA had no significant effect on corneal graft survival, Alalwani et al. [28] found that 2% CsA in eye-drops could effectively inhibit corneal rejection. In agreement with these authors, we also found that topical application of 1% CsA in eye-drops effectively prolonged corneal graft survival.

We do not find any statistically significant difference in the percentage of CD4+ T cells in PBLs among the five groups. The reason for this may be that the systemic absorption after topical drug application is limited. The flow cytometric analysis showed that there was a statistically significant difference in the percentage of CD4+ T cells in the cervical lymph nodes when using topical 0.5% FTY720 (versus untreated animals) and is in agreement with studies showing that FTY720 induced inhibition of T cells egress from lymphoid organs due to modification of S1P [13,15]. Similar to that of the systemic therapy, topical 0.5% FTY720 reduced egress of lymphocytes from the lymph nodes and stimulated CD4+ T cells of the cornea to home to the cervical lymph nodes. And as is commonly known, CD4+T cells are the most important T cell population involved in corneal allograft rejection. Therefore, we suggest that topical 0.5% FTY720 prolongs corneal allograft survival through a decrease in the number of CD4+ T cells in the cornea.

We found that topical 0.5% FTY720 significantly enhances the percentage of CD4+CD25+foxp3+ T cells in the cervical lymphoid nodes followed by the change of the CD4+T cell distribution. This may be because FTY720 can increase the percentage of Treg cells in the lymph nodes by means of the S1P receptor. It has been reported that CD4+CD25+ Treg cells expressed lower levels of mRNA for *S1P1* and *S1P4* receptors and demonstrated a reduced chemotactic response to S1P [34]. Additionally, FTY720 can be phosphorylated in vivo by SphK2 to FTY720-P, which acts as a potent S1P receptor agonist [14]. These results are in line with some studies showing that systemic FTY720 can significantly increase the percentage of Treg cells [35,36]. CD4+CD25+Foxp3+ Treg cells are a functionally distinct subset of T cells with suppressive ability and prevent allograft rejection [9,10]. Matsuoka et al. [37] found that CD4+ lymphopenia was a critical factor in Treg cell homeostasis, and that prolonged imbalance of Treg cell homeostasis resulted in a loss of tolerance and significant clinical disease manifestations. Moreover, some studies had shown that homing of Treg cells into the draining lymph nodes was required for the suppressive function of these cells [38,39]. Therefore, the specific suppressive function of topical 0.5% FTY720 on corneal transplantation can be explained by significantly increasing the percentage of Treg cells in the cervical lymph nodes. We did not find any statistical difference in the percentage of CD4+ T and Treg cells in the 1% CsA group, confirming that topical 1% CsA can not affect the distribution of these cells.

To investigate further the mechanism by which topical 0.5% FTY720 prolongs corneal allograft survival, we analyzed the intra-graft mRNA gene expression of cytokines. Although topical 0.5% FTY720 can change the Treg cell distribution, we found that *TGF-β1* mRNA expression (and corneal immunohistochemical staining) in this group was higher than that in the other groups. Several studies have found that Treg cells can produce TGF-β1 and Treg cell suppression was associated with TGF-β1 [40,41]. Based on these results, we suggest that topical 0.5% FTY720 may significantly enhance the immune function of Treg cells by increasing levels of TGF-β1 in the cornea. Moreover, we also found that *Foxp3* mRNA expression after topical 0.5% FTY720 is slightly higher (albeit not significantly) compared with the other groups. It has been clearly shown that the forkhead family transcription factor, Foxp3, is critically important for the development and function of regulatory T cells [42]. Therefore, we further hypothesize that topical 0.5% FTY20 significantly enhances the immune activity of corneal Treg cells. The enhanced suppressive immune function of Treg cells can reinforce the inhibition in the rejection of allogeneic corneal transplants.

We found a significant decrease in *IL-2* and *IFN-γ* mRNA expression after topical application of 1% CsA, which was also seen as reduced immunohistochemical staining in corneal grafts compared with the control group. These results are concordant with the other studies showing that CsA suppresses T cell proliferation by inhibiting the synthesis of IL-2 and IFN-γ [43-45].

In summary, our results confirm that topical 0.5% FTY720 or 1% CsA can effectively prolong mouse allogeneic corneal graft survival for a one-month period. Topical 0.5% FTY720 and 1% CsA have different pathways involved in the suppression of corneal graft rejection. FTY720 increases the percentage of CD4+ T cells and a raises the percentage of Treg cells in cervical lymph nodes. In addition, topical 0.5% FTY720 enhances the immune function of Treg cells by increasing *TGF-β1* mRNA and protein expression, and decreaseing CD4+ T cell infiltration into the corneal allograft. Unlike topical 0.5% FTY720, topical 1% CsA inhibits CD4+ T cell proliferation and decreases the expression of IL-2 and IFN-γ in the corneas. However, we did not find that topical FTY720 and CsA completely inhibited the rejection of the corneal allograft.

## References

[1] Whitcher JP, Srinivasan M, Upadhyay MP (2001). Corneal blindness: a global perspective.. Bull World Health Organ.

[2] Rocha G, Deschenes J, Rowsey JJ (1998). The immunology of corneal graft rejection.. Crit Rev Immunol.

[3] Williams KA, Lowe M, Bartlett C, Kelly TL, Coster DJ, All C (2008). Risk factors for human corneal graft failure within the Australian corneal graft registry.. Transplantation.

[4] Alldredge OC, Krachmer JH (1981). Clinical types of corneal transplant rejection. Their manifestations, frequency, preoperative correlates, and treatment.. Arch Ophthalmol.

[5] Maguire MG, Stark WJ, Gottsch JD, Stulting RD, Sugar A, Fink NE, Schwartz A (1994). Risk factors for corneal graft failure and rejection in the collaborative corneal transplantation studies. Collaborative Corneal Transplantation Studies Research Group.. Ophthalmology.

[6] Williams KA, Roder D, Esterman A, Muehlberg SM, Coster DJ (1992). Factors predictive of corneal graft survival. Report from the Australian Corneal Graft Registry.. Ophthalmology.

[7] Panda A, Vanathi M, Kumar A, Dash Y, Priya S (2007). Corneal graft rejection.. Surv Ophthalmol.

[8] Niederkorn JY (2007). Immune mechanisms of corneal allograft rejection.. Curr Eye Res.

[9] Chauhan SK, Saban DR, Lee HK, Dana R (2009). Levels of Foxp3 in regulatory T cells reflect their functional status in transplantation.. J Immunol.

[10] Cunnusamy K, Paunicka K, Reyes N, Yang W, Chen PW, Niederkorn JY (2010). Two different regulatory T cell populations that promote corneal allograft survival.. Invest Ophthalmol Vis Sci.

[11] Inoue K, Kimura C, Amano S, Sato T, Fujita N, Kagaya F, Kaji Y, Tsuru T, Araie M (2001). Long-term outcome of systemic cyclosporine treatment following penetrating keratoplasty.. Jpn J Ophthalmol.

[12] Shimazaki J, Den S, Omoto M, Satake Y, Shimmura S, Tsubota K. (2011). Prospective, randomized study of the efficacy of systemic cyclosporine in high-risk corneal transplantation.. American Journal of Ophthalmology.

[13] Suzuki S, Enosawa S, Kakefuda T, Shinomiya T, Amari M, Naoe S, Hoshino Y, Chiba K (1996). A novel immunosuppressant, FTY720, with a unique mechanism of action, induces long-term graft acceptance in rat and dog allotransplantation.. Transplantation.

[14] Hanessian S, Charron G, Billich A, Guerini D (2007). Constrained azacyclic analogues of the immunomodulatory agent FTY720 as molecular probes for sphingosine 1-phosphate receptors.. Bioorg Med Chem Lett.

[15] Brinkmann V, Davis MD, Heise CE, Albert R, Cottens S, Hof R, Bruns C, Prieschl E, Baumruker T, Hiestand P, Foster CA, Zollinger M, Lynch KR (2002). The immune modulator FTY720 targets sphingosine 1-phosphate receptors.. J Biol Chem.

[16] Salvadori M, Budde K, Charpentier B, Klempnauer J, Nashan B, Pallardo LM, Eris J, Schena FP, Eisenberger U, Rostaing L, Hmissi A, Aradhye S, FTY720 0124 Study Group (2006). FTY720 versus MMF with cyclosporine in de novo renal transplantation: a 1-year, randomized controlled trial in Europe and Australasia.. Am J Transplant.

[17] Li Q, Li F (2010). Effects of different dose of FTY720 on lymphocyte cell cycle arrest in cardiac transplantation model of rats.. Immunopharmacol Immunotoxicol.

[18] Sedláková K, Muckersie E, Robertson M, Filipec M, Forrester JV (2005). FTY720 in corneal concordant xenotransplantation.. Transplantation.

[19] Mayer K, Birnbaum F, Reinhard T, Reis A, Braunstein S, Claas F, Sundmacher R (2004). FTY720 prolongs clear corneal allograft survival with a differential effect on different lymphocyte populations.. Br J Ophthalmol.

[20] Zhang EP, Muller A, Ignatius R, Hoffmann F (2003). Significant prolongation of orthotopic corneal-graft survival in FTY720-treated mice.. Transplantation.

[21] Cohen JA, Barkhof F, Comi G, Hartung HP, Khatri BO, Montalban X, Pelletier J, Capra R, Gallo P, Izquierdo G, Tiel-Wilck K, de Vera A, Jin J, Stites T, Wu S, Aradhye S, Kappos L, Group TS (2010). Oral fingolimod or intramuscular interferon for relapsing multiple sclerosis.. N Engl J Med.

[22] Zhang EP, Schrunder S, Hoffmann F (1996). Orthotopic corneal transplantation in the mouse–a new surgical technique with minimal endothelial cell loss.. Graefes Arch Clin Exp Ophthalmol.

[23] Larkin DF, Calder VL, Lightman SL (1997). Identification and characterization of cells infiltrating the graft and aqueous humour in rat corneal allograft rejection.. Clin Exp Immunol.

[24] Xie Y, Sun HX, Li D (2009). Platycodin D is a potent adjuvant of specific cellular and humoral immune responses against recombinant hepatitis B antigen.. Vaccine.

[25] Zhang JL, Sun DJ, Hou CM, Wei YL, Li XY, Yu ZY, Feng JN, Shen BF, Li Y, Xiao H (2010). CD3 mAb treatment ameliorated the severity of the cGVHD-induced lupus nephritis in mice by up-regulation of Foxp3+ regulatory T cells in the target tissue: kidney.. Transpl Immunol.

[26] Küchle M, Cursiefen C, Nguyen NX, Langenbucher A, Seitz B, Wenkel H, Martus P, Naumann GO (2002). Risk factors for corneal allograft rejection: intermediate results of a prospective normal-risk keratoplasty study.. Graefes Arch Clin Exp Ophthalmol.

[27] Xie L, Shi W, Wang Z, Bei J, Wang S (2001). Prolongation of corneal allograft survival using cyclosporine in a polylactide-co-glycolide polymer.. Cornea.

[28] Alalwani H, Omer Saleh B, Rocher N, Renard G, Bourges JL, Legeais JM (2010). Advantages and limits of multiple grafts (third keratoplasty) under local cyclosporin 2%.. J Fr Ophtalmol.

[29] Bourges JL, Lallemand F, Agla E, Besseghir K, Dumont JM, BenEzra D, Gurny R, Behar-Cohen F (2006). Evaluation of a topical cyclosporine A prodrug on corneal graft rejection in rats.. Mol Vis.

[30] Poon A, Constantinou M, Lamoureux E, Taylor HR (2008). Topical Cyclosporin A in the treatment of acute graft rejection: a randomized controlled trial.. Clin Experiment Ophthalmol.

[31] Sinha R, Jhanji V, Verma K, Sharma N, Biswas NR, Vajpayee RB (2010). Efficacy of topical cyclosporine A 2% in prevention of graft rejection in high-risk keratoplasty: a randomized controlled trial.. Graefes Arch Clin Exp Ophthalmol.

[32] Unal M, Yucel I (2008). Evaluation of topical ciclosporin 0.05% for prevention of rejection in high-risk corneal grafts.. Br J Ophthalmol.

[33] Premenko-Lanier M, Moseley NB, Pruett ST, Romagnoli PA, Altman JD (2008). Transient FTY720 treatment promotes immune-mediated clearance of a chronic viral infection.. Nature.

[34] Sawicka E, Dubois G, Jarai G, Edwards M, Thomas M, Nicholls A, Albert R, Newson C, Brinkmann V, Walker C (2005). The sphingosine 1-phosphate receptor agonist FTY720 differentially affects the sequestration of CD4+/CD25+ T-regulatory cells and enhances their functional activity.. J Immunol.

[35] Commodaro AG, Peron JP, Lopes CT, Arslanian C, Belfort R, Rizzo LV, Bueno V (2010). Evaluation of experimental autoimmune uveitis in mice treated with FTY720.. Invest Ophthalmol Vis Sci.

[36] Sehrawat S, Rouse BT (2008). Anti-inflammatory effects of FTY720 against viral-induced immunopathology: role of drug-induced conversion of T cells to become Foxp3+ regulators.. J Immunol.

[37] Matsuoka K, Kim HT, McDonough S, Bascug G, Warshauer B, Koreth J, Cutler C, Ho VT, Alyea EP, Antin JH, Soiffer RJ, Ritz J (2010). Altered regulatory T cell homeostasis in patients with CD4+ lymphopenia following allogeneic hematopoietic stem cell transplantation.. J Clin Invest.

[38] Ochando JC, Yopp AC, Yang Y, Garin A, Li Y, Boros P, Llodra J, Ding Y, Lira SA, Krieger NR, Bromberg JS (2005). Lymph node occupancy is required for the peripheral development of alloantigen-specific Foxp3+ regulatory T cells.. J Immunol.

[39] Schneider MA, Meingassner JG, Lipp M, Moore HD, Rot A (2007). CCR7 is required for the in vivo function of CD4+ CD25+ regulatory T cells.. J Exp Med.

[40] Daley SR, Ma J, Adams E, Cobbold SP, Waldmann H (2007). A key role for TGF-beta signaling to T cells in the long-term acceptance of allografts.. J Immunol.

[41] Lu L, Ma J, Wang X, Wang J, Zhang F, Yu J, He G, Xu B, Brand DD, Horwitz DA, Shi W, Zheng SG (2010). Synergistic effect of TGF-beta superfamily members on the induction of Foxp3+ Treg.. Eur J Immunol.

[42] Ziegler SF (2006). FOXP3: of mice and men.. Annu Rev Immunol.

[43] Kobayashi T, Momoi Y, Iwasaki T. (2007). Cyclosporine A inhibits the mRNA expressions of IL-2, IL-4 and IFN-gamma, but not TNF-alpha, in canine mononuclear cells.. J Vet Med Sci.

[44] Tian L, Stepkowski SM, Qu X, Wang ME, Wang M, Yu J, Kahan BD (1997). Cytokine mRNA expression in tolerant heart allografts after immunosuppression with cyclosporine, sirolimus or brequinar.. Transpl Immunol.

[45] Fruman DA, Klee CB, Bierer BE, Burakoff SJ (1992). Calcineurin phosphatase activity in T lymphocytes is inhibited by FK 506 and cyclosporin A.. Proc Natl Acad Sci USA.

